# Role of endothelial hyaluronan in peritoneal membrane transport and disease conditions during peritoneal dialysis

**DOI:** 10.1038/s41598-024-58148-x

**Published:** 2024-03-28

**Authors:** Keisuke Kamiya, Naoyuki Hatayama, Mitsuhiro Tawada, Akimasa Asai, Mai Yamauchi, Hiroshi Kinashi, Shunnosuke Kunoki, Makoto Yamaguchi, Masashi Mizuno, Yasuhiro Suzuki, Masataka Banshodani, Takuji Ishimoto, Munekazu Naito, Hideki Kawanishi, Yasuhiko Ito

**Affiliations:** 1 Department of Nephrology and Rheumatology, Aichi Medical University, 1-1 Karimata, Yazako, Nagakute City, Aichi, 480-1195 Japan; 2 Department of Anatomy, Aichi Medical University School of Medicine, Nagakute, Aichi Japan; 3 Department of Nephrology, Nagoya University Graduate School of Medicine, Nagoya, Aichi Japan; 4 Department of Nephrology, Nippon Medical School, Tokyo, Japan; 5 Department of Surgery and Artificial Organs, Akane-Foundation, Tsuchiya General Hospital, Hiroshima, Japan

**Keywords:** Glycocalyx, Hyaluronan, Peritoneal transport, Macromolecules, Protein leakage, EPS, Nephrology, Renal replacement therapy

## Abstract

Peritoneal membrane dysfunction in peritoneal dialysis (PD) is primarily attributed to angiogenesis; however, the integrity of vascular endothelial cells can affect peritoneal permeability. Hyaluronan, a component of the endothelial glycocalyx, is reportedly involved in preventing proteinuria in the normal glomerulus. One hypothesis suggests that development of encapsulating peritoneal sclerosis (EPS) is triggered by protein leakage due to vascular endothelial injury. We therefore investigated the effect of hyaluronan in the glycocalyx on peritoneal permeability and disease conditions. After hyaluronidase-mediated degradation of hyaluronan on the endothelial cells of mice, macromolecules, including albumin and β2 microglobulin, leaked into the dialysate. However, peritoneal transport of small solute molecules was not affected. Pathologically, hyaluronan expression was diminished; however, expression of vascular endothelial cadherin and heparan sulfate, a core protein of the glycocalyx, was preserved. Hyaluronan expression on endothelial cells was studied using 254 human peritoneal membrane samples. Hyaluronan expression decreased in patients undergoing long-term PD treatment and EPS patients treated with conventional solutions. Furthermore, the extent of hyaluronan loss correlated with the severity of vasculopathy. Hyaluronan on endothelial cells is involved in the peritoneal transport of macromolecules. Treatment strategies that preserve hyaluronan in the glycocalyx could prevent the leakage of macromolecules and subsequent related complications.

## Introduction

The decline in ultrafiltration capacity associated with high peritoneal transport seen after long-term peritoneal dialysis (PD) is a major cause of PD discontinuation^1,2^. Several studies have indicated that a higher peritoneal solute transport rate is also associated with decreased survival of PD patients^1,3^. The major pathological findings of increased peritoneal permeability in PD were thought to be due to angiogenesis associated with fibrosis^4,5^. However, recent studies have indicated that despite an increase in the number of blood vessels, peritoneal permeability does not increase after long-term PD treatment with low-glucose degradation products (GDPs), which are pH-neutral solutions^6^. This suggest that factors other than the number of blood vessels affect peritoneal permeability. The integrity of blood vessel structures such as the endothelial glycocalyx may affect peritoneal permeability.

Vascular endothelial cells are covered by the glycocalyx, a bioactive gel-like layer consisting of heparan sulfate, hyaluronan, chondroitin sulfate, and associated proteins^7–11^. The endothelial glycocalyx of blood vessels is known to play roles in maintaining a negative charge, regulating coagulation, and controlling microvascular permeability^10,12–14^. The glomerular endothelial glycocalyx, and particularly its component hyaluronan, has recently been shown to play a role in protecting against the development of proteinuria and structural damage^15–17^; however, the role of hyaluronan in the glycocalyx in maintaining peritoneal membrane permeability remains unclear.

The present study investigated the roles of hyaluronan in the glycocalyx in maintaining peritoneal membrane permeability and in disease conditions during PD. As one hypothesis suggests that encapsulating peritoneal sclerosis (EPS) development is triggered by protein leakage due to vascular endothelial injury^18,19^, we digested hyaluronan in the peritoneal membrane in mice and examined the expression of hyaluronan following peritoneal vascular injury, so-called vasculopathy, in human studies.

## Methods

### Animals

Eight-week-old male C57BL/6JJmsSlc mice (Japan SLC, Shizuoka, Japan) were used for in vivo experiments. Animals were housed and maintained under conventional standard laboratory conditions with free access to food and water. The Institutional Animal Care and Usage Committee of Aichi Medical University (Nagakute, Japan) approved the experimental protocol (approval numbers 2019–76 and 2022–9).

### Measurement of peritoneal solute transport rate

Mouse peritoneal membrane function was assessed using a peritoneal equilibration test (PET), as previously described^20,21^. A total of 2000 μl of 4.25% glucose peritoneal dialysis fluid (PDF; Dianeal PD-4 4.25; Baxter, Tokyo, Japan) was administered into the peritoneal cavity. After 2 h, all dialysate remaining in the peritoneal cavity was collected, and blood samples were obtained. The concentrations of urea nitrogen and glucose in serum and dialysate were measured using enzymatic methods and ultraviolet absorption spectrophotometry, respectively. BCA Protein Assay (Thermo Fisher Scientific, Waltham, MA) and LBIS Mouse Urinary-Albumin assay kit (Wako Pure Chemicals, Osaka, Japan) were used to measure the protein concentration and albumin leakage in the dialysate, respectively. The concentrations of serum hyaluronan and serum and dialysate β2 microglobulin (β2MG) were measured using a hyaluronan enzyme-linked immunosorbent assay (ELISA) kit (PG Research, Tokyo, Japan)^15^ and mouse β2MG ELISA kit (Abcam, Cambridge, UK), respectively. The dialysate-to-plasma β2MG ratio (D/P β2MG) and ratio of dialysate glucose at 2 h of dwell time to dialysis glucose at baseline (D2/D0 glucose) were also determined.

### Determination of the effect of hyaluronidase on peritoneal membrane transport rate in mice

**Figure 1:**
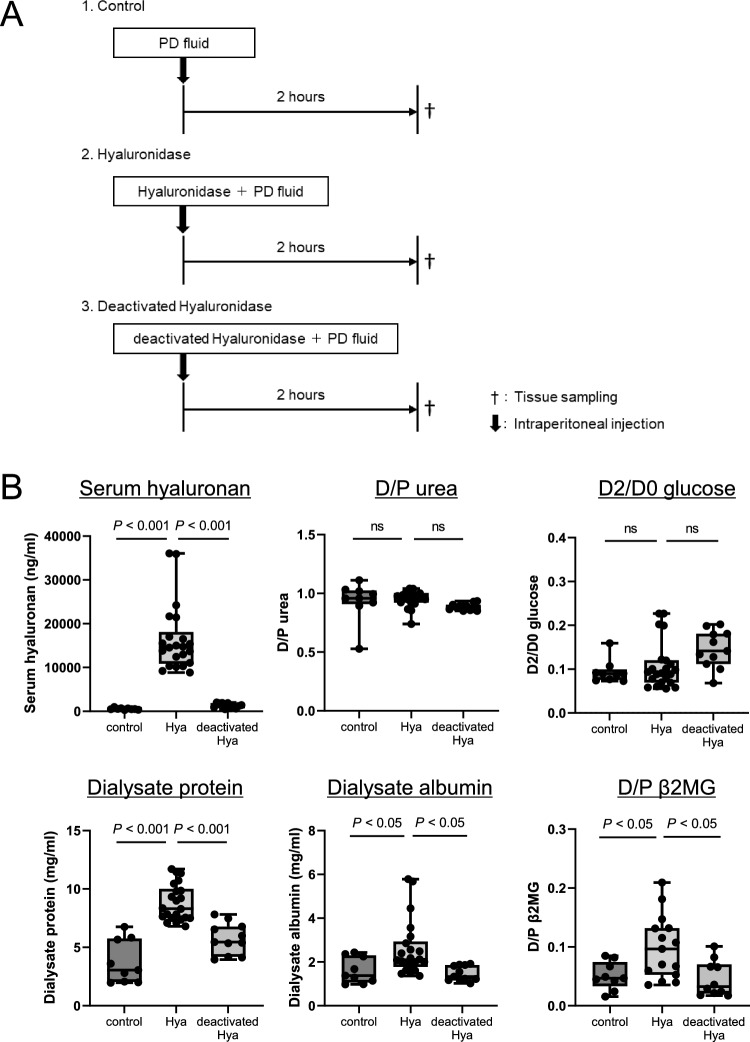

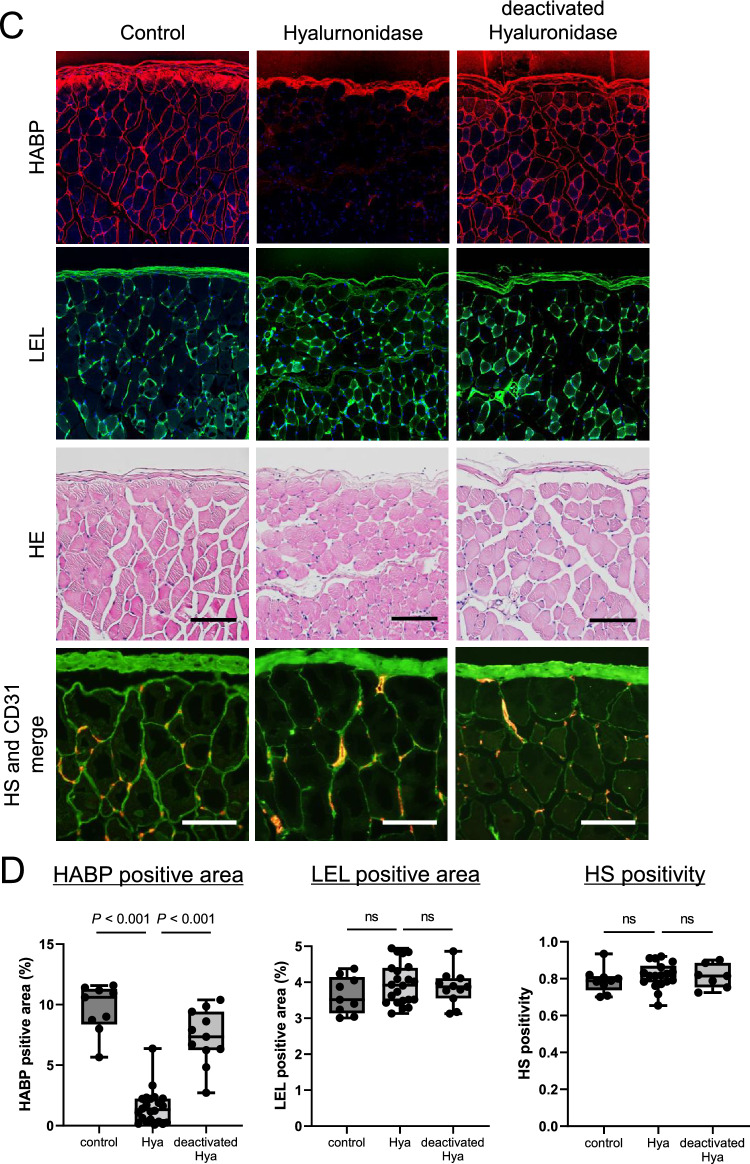
Effect of hyaluronidase on peritoneal permeability and vascular endothelial cells. (**A**): Mice were intraperitoneally injected with 2 ml of PD fluid (PDF) containing 20 mg hyaluronidase (Hyaluronidase group, n = 22), 2 ml of PDF containing 20 mg deactivated hyaluronidase (Deactivated-hyaluronidase group, n = 11), or 2 ml of PDF alone (Control group, n = 9). Two hours after injection, mice were sacrificed. Peritoneal membrane function and histochemistry were assessed. (**B**): In the Hyaluronidase group, serum hyaluronan levels, protein and albumin leakage to the dialysate, and D/P β2MG ratio were significantly increased; however, D/P urea and D2/D0 glucose were not changed. (**C,D**): Hyaluronan expression assessed by HABP staining was decreased by hyaluronidase treatment but not by deactivated hyaluronidase. LEL-positive area and HS positivity in CD31-positive vessels were not different between the Hyaluronidase group and Deactivated-hyaluronidase group. *Hya* hyaluronidase group, *deactivated Hya* deactivated-hyaluronidase group, *D/P urea* dialysate-to-plasma urea ratio, *D2/D0 glucose* dialysate 2 h-to-dialysate 0 h glucose reabsorption ratio, *D/P β2MG* dialysate-to-plasma beta-2 microglobulin ratio, *HABP* hyaluronan-binding protein, *LEL Lycopersicon esculentum* lectin, *HS* heparan sulfate, *n.s.* not significant. Scale bars (HABP, LEL, and HE) = 100 μm. Scale bars (HS and CD31) = 50 μm.

The effect of hyaluronidase on peritoneal membrane function was assessed using a PET. Briefly, mice were intraperitoneally injected with 2 ml of PDF alone (Control group, n = 9), 2 ml of PDF containing 20 mg hyaluronidase (Sigma-Aldrich, St. Louis, MO) (Hyaluronidase group, n = 22), or 2 ml of PDF containing 20 mg hyaluronidase deactivated by heating at 90 °C for 30 min (Deactivated-hyaluronidase group, n = 11) (Fig. 1A). Two hours after injection, the mice were sacrificed, and PDF, blood, and peritoneal tissues were harvested for subsequent histochemical analysis and measurement of urea, glucose, protein, β2MG, and hyaluronan to determine the peritoneal transport rate (Fig. 1).

**Figure 2:**
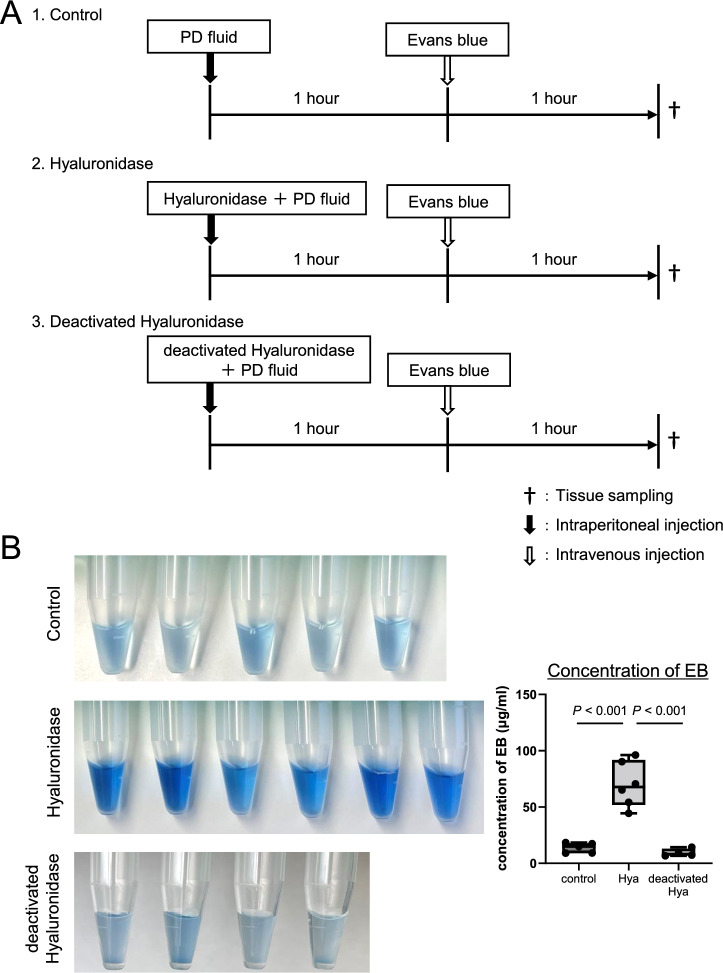
Effect of hyaluronidase on leakage of Evans blue. (**A**): In order to assess protein leakage into the dialysate, mice of the three groups (Hyaluronidase group, n = 6, Deactivated-hyaluronidase group, n = 4, Control group, n = 5) were administered Evans blue (EB) via the ophthalmic vein at 1 h. One hour later, PDF was obtained, and the absorbance was measured using an absorptiometer. (**B**): Significant leakage of EB into the dialysate was observed in the Hyaluronidase group. *Hya* hyaluronidase group, *deactivated Hya* deactivated-hyaluronidase group.

Evans blue (EB), a pigment with high affinity for blood albumin, leaks out of the capillaries together with albumin if vascular permeability is increased^22,23^. To evaluate protein leakage to the dialysate, PDF was administered according to the same protocol used for the three above-mentioned groups (Hyaluronidase group, n = 6; Deactivated-hyaluronidase group, n = 4; Control group, n = 5), and then 1 h later, EB (Wako Pure Chemicals) dissolved in 0.1 ml of saline was intravenously administered via the ophthalmic vein. Two hours after injection of PDF, PDF was recovered, and the absorbance was measured using an absorptiometer (620 nm) (Fig. 2). The EB concentration was calculated according to a calibration curve.

**Figure 3:**
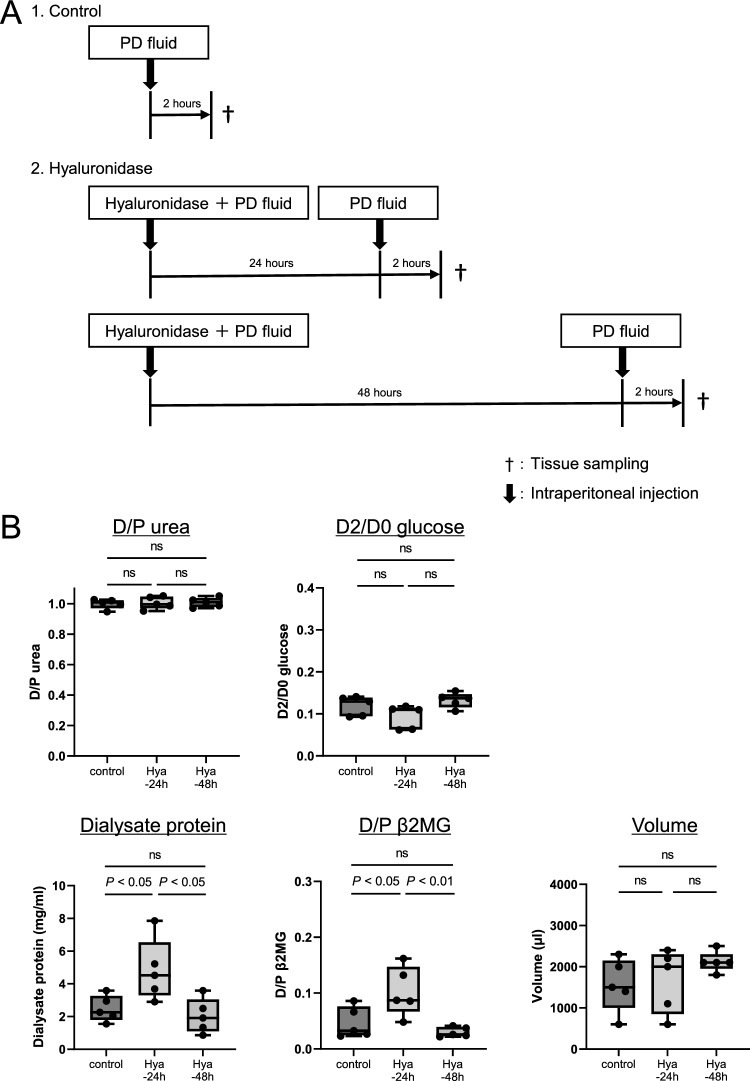

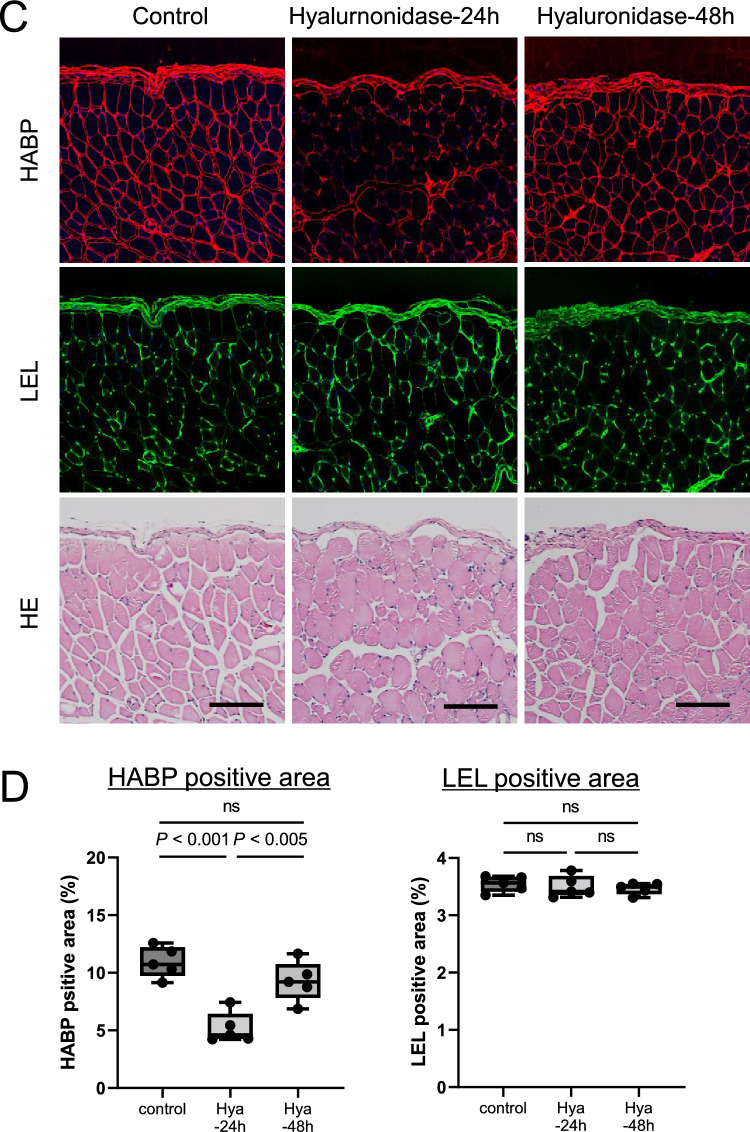
Monitoring of hyaluronan expression and peritoneal membrane transport after intraperitoneal administration of hyaluronidase. (**A**): Peritoneal membrane function and pathology were assessed at 24 and 48 h after administration of hyaluronidase. PET was conducted by intraperitoneal injection of 4.25% glucose PDF, with sacrifice of mice 2 h later. (**B**): Degree of protein leakage into dialysate and peritoneal membrane transport of β2MG were decreased and returned to the control levels by 48 h after hyaluronidase administration. (**C,D**): Hyaluronan expression assessed by HABP staining was still low 24 h after hyaluronidase treatment; however, it recovered to the control level by 48 h. Vascular endothelial cells assessed by LEL staining were not changed at 24 and 48 h. *HABP* hyaluronan-binding protein, *LEL Lycopersicon esculentum* lectin, *n.s.* not significant. Scale bars = 100 μm.

In order to monitor peritoneal membrane function (Fig. 3A), the PET and pathologic evaluations were carried out at 24 and 48 h after administration of PDF with 20 mg hyaluronidase. The PET was conducted by intraperitoneal injection of PDF at 24 or 48 h, and 2 h later, the animals were sacrificed, and samples (PDF and tissues) were collected.

### Histology and immunohistochemistry

A portion of the peritoneal tissue was fixed in Maskedform (Japan Tanner, Osaka, Japan), a commercially available formalin fixative, and embedded in paraffin using standard methods. Four-micrometer-thick sections were stained with hematoxylin and eosin (HE) for routine pathological evaluation. Peritoneal hyaluronan expression was visualized using biotin-labeled recombinant human hyaluronan-binding protein (HABP) (Hokudo, Sapporo, Japan) on paraffin-embedded sections according to the manufacturer’s instructions^15^. To identify the capillaries on paraffin sections, staining for tomato-derived *Lycopersicon esculentum* lectin (LEL), a marker of endothelial cells, was conducted^9,15,24^. For double-staining of CD31 and vascular endothelial cadherin (VE-cadherin) on mouse frozen tissues, anti–VE cadherin antibody (Bioss Antibodies, Woburn, MA) was applied, and sections were then incubated with anti-mouse CD31 antibodies (Merck, Darmstadt, Germany). After washing with PBS, sections were incubated sequentially with Alexa Fluor 555–labeled goat anti-rat IgG and Alexa Fluor 488–labeled goat anti-rabbit IgG as secondary antibodies.

For double-staining of heparan sulfate (HS) and CD31 on mouse frozen tissues, sections were incubated sequentially with anti-mouse CD31 antibody and Alexa Fluor 555–labeled goat anti-rat IgG. After washing with PBS, anti-HS antibody (US Biological, Salem, MA) labeled with FITC using a labeling kit (DOJINDO, Kumamoto, Japan) was applied. VE-cadherin and HS positivity on blood vessels were calculated based on the number of VE-cadherin– or HS-positive vessels in mice peritoneum divided by the number of CD31-positive vessels on the same section, respectively. Immunohistochemistry was assessed using confocal microscopy (FV-3000; Olympus, Tokyo, Japan) and recorded using FV31S-SW software (Olympus). Details of antibodies and ELISA methods are listed in Supplementary Table S1.

### Expression of hyaluronan on human peritoneal membrane biopsied samples

**Figure 4:**
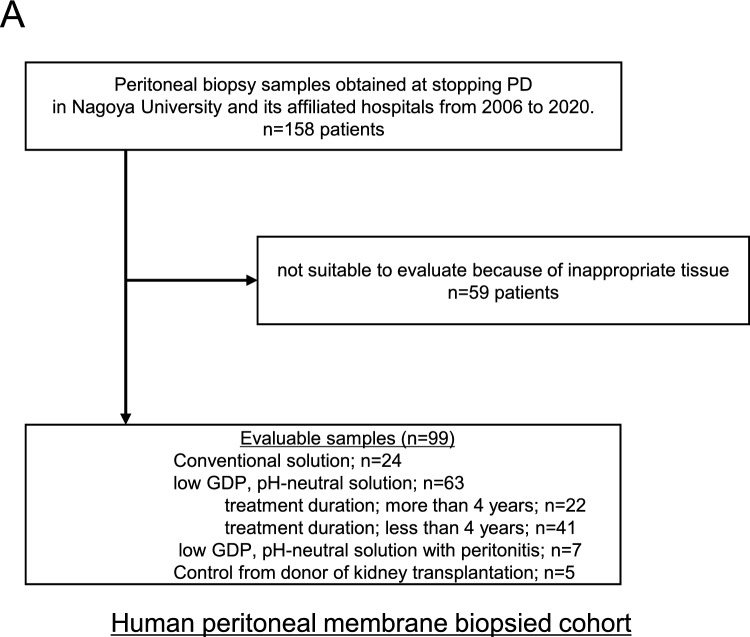

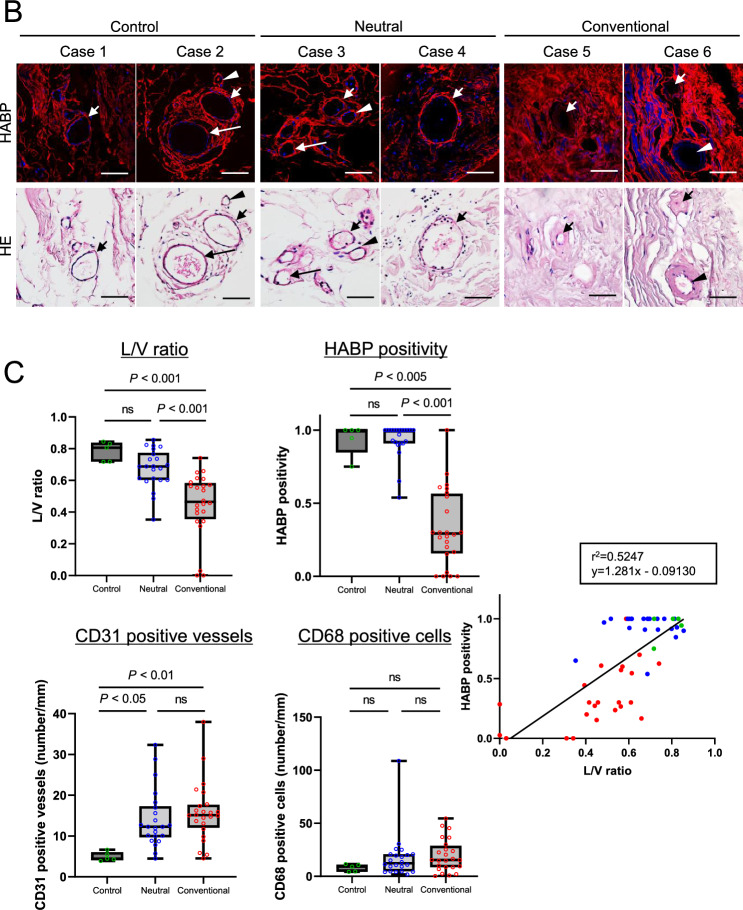

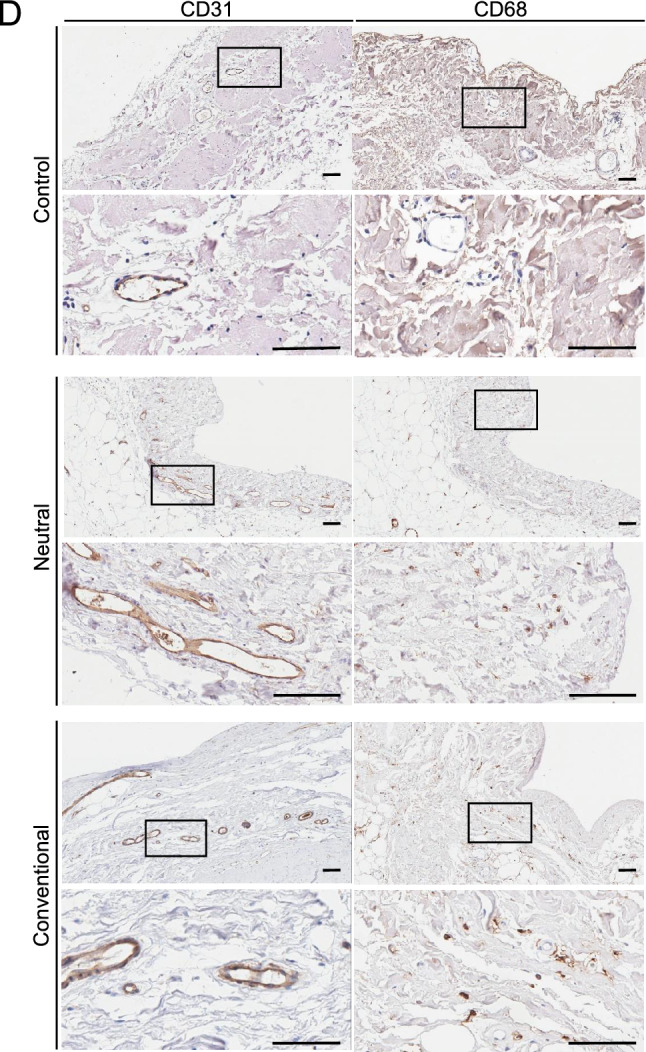
Expression of hyaluronan in human peritoneal membrane treated with conventional solutions or low-GDP, pH-neutral solutions. (**A**) Flow diagram of the study population. (Human peritoneal membrane biopsied cohort). (**B**) HABP and HE staining. (**C**) Differences in L/V ratio, HABP positivity, CD31-positive vessels, and CD68-positive cells. (**D**) Expression on CD31-positive vessels and CD68-positive macrophages. Two representative cases are shown in each group. HE staining was conducted after HABP staining on the same sections. Arrows and arrowheads indicate the same vessels in each case. Expression of hyaluronan assessed by HABP positivity was decreased in association with lower L/V ratio in the conventional solution group. There was a good correlation between L/V ratio and HABP positivity (r^2^ = 0.524, *p* < 0.0001). There was no significant difference in the number of CD31-positive vessels and CD68-positive macrophages between the two solution groups. Control: (green circle); Conventional: conventional solution group (red circle); Neutral: low-GDP, pH-neutral solution group (blue circle); L/V ratio: luminal diameter to vessel diameter; *HABP* hyaluronan-binding protein, *LEL Lycopersicon esculentum* lectin, *HS* heparan sulfate, *n.s.* not significant. Scale bars = 100 μm.

**Figure 5:**
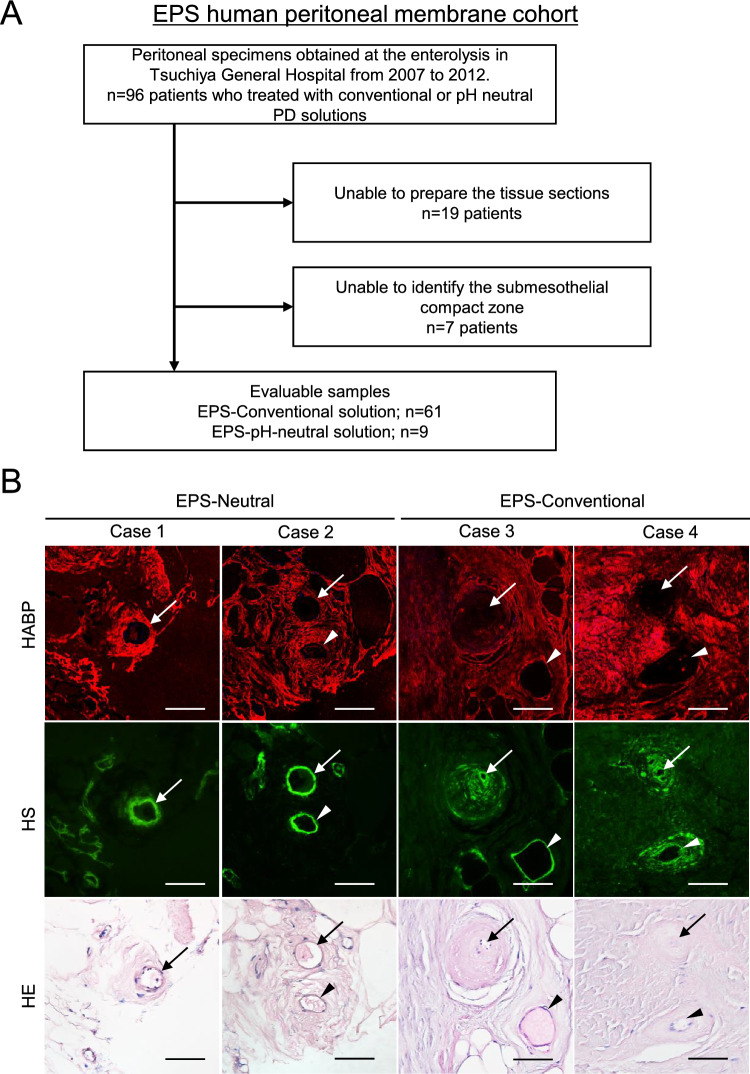

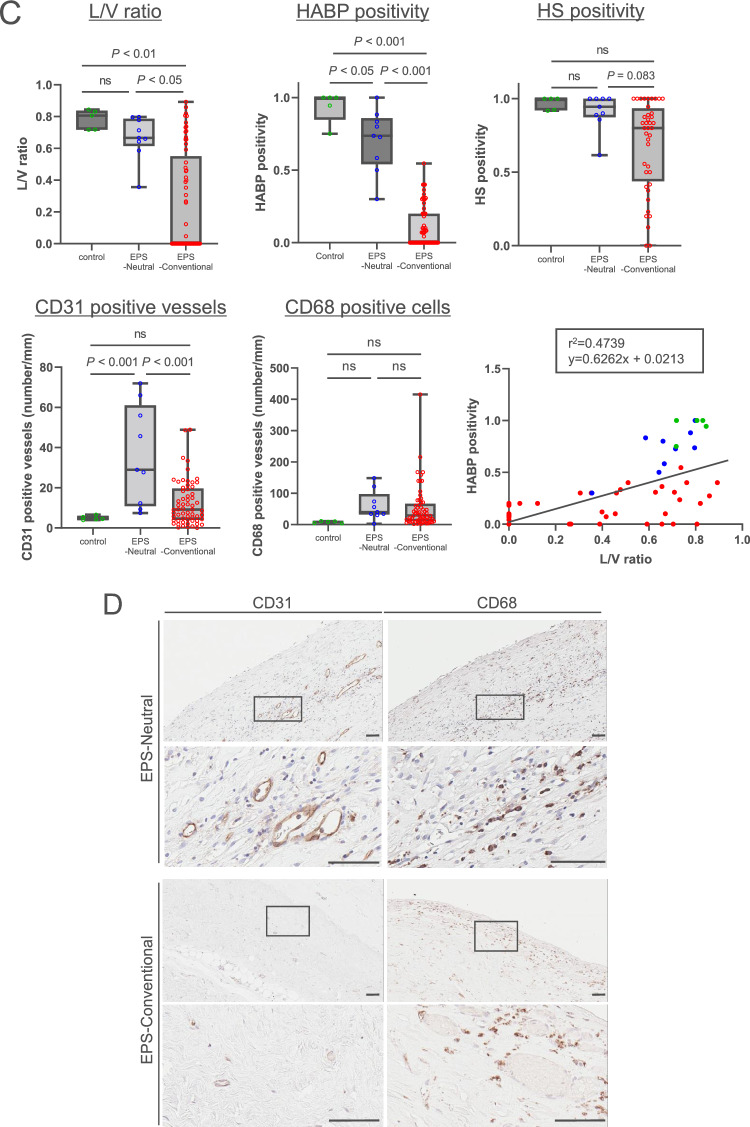
Expression of hyaluronan in EPS human peritoneal membrane. (**A**)Flow diagram of the study population. (EPS human peritoneal membrane cohort). (**B**) HABP, HS, and HE staining. (**C**) Differences in L/V ratio, HABP and HS positivity, CD31-positive vessels, and CD68-positive cells. (**D**) Expression on CD31-positive vessels and CD68-positive macrophages. Two representative cases are shown in each group. HE staining was conducted after HABP staining on the same sections. Arrows and arrowheads indicate the same vessels in each case. Expression of hyaluronan assessed by HABP positivity was decreased in association with lower L/V ratio in EPS cases treated with conventional solution. HS was more preserved than hyaluronan, as assessed by HABP staining. A correlation between L/V ratio and HABP positivity was observed (r^2^ = 0.474, *p* < 0.0001). The number of CD31-positive vessels was higher in the EPS-neutral group. Control: (green circle); EPS-Conventional: EPS cases treated with conventional solution during PD (red circle); EPS-Neutral: EPS cases treated with low-GDP, pH-neutral solution during PD (blue circle); L/V ratio: luminal diameter to vessel diameter; *HABP* hyaluronan-binding protein, *LEL Lycopersicon esculentum* lectin, *HS* heparan sulfate, *n.s.* not significant. Scale bars = 100 μm.

Human studies were conducted according to the Declaration of Helsinki and approved by the Tsuchiya General Hospital Ethics Committee (approval number E160530-1) and the Ethics Committee for Human Research of the Faculty of Medicine at Nagoya University (approval number 299-7). Informed consent was obtained from all patients. The study involved two peritoneal membrane pathology cohorts consisting of a total of 254 patients. One peritoneal membrane cohort included biopsies taken at cessation of PD (human peritoneal membrane biopsied cohort, n = 158) at Nagoya University Hospital (Nagoya, Japan) and hospitals affiliated with Nagoya University (Fig. 4A). The peritoneal membrane EPS cohort samples were taken at the time of EPS surgical enterolysis (EPS human peritoneal membrane cohort, n = 96) at Tsuchiya General Hospital (Hiroshima, Japan) (Fig. 5A). All patients were Japanese and over 18 years of age.

In the EPS human peritoneal membrane cohort, a total of 158 biopsy samples were screened, and 99 cases were evaluated. We excluded 59 samples that were judged unsuitable because of insufficient tissue size, lack of peritoneal surface membrane, or difficulty cutting the tissues due to calcification, according to the methods of previous reports^18,25,26^. Patients treated with low GDPs, which are pH-neutral solutions (pH-neutral–solution group), were divided into two groups by treatment duration of more (n = 22) or less (n = 41) than 4 years. Peritoneal tissues after PD treatment with conventional solutions (Conventional-solution group), those after treatment with neutral solution (pH-neutral–solution group), and tissues exhibiting peritonitis (n = 7) were taken at catheter removal for stopping PD according to procedures described previously (Fig. 4A)^18,19^. Peritoneal tissues of EPS cases (Fig. 5A) after the use of conventional solutions (EPS-conventional: n = 61) and EPS cases after use of low-GDP, pH-neutral solutions (EPS-pH neutral: n = 9) were taken from the anterior abdominal wall at the time of surgical enterolysis. Five control tissues were taken from kidney transplantation donors. Flow diagrams of each study are shown in Figs. 4A and 5A. Clinical data, including the primary kidney disease present, duration of PD treatment, occurrence rate of peritonitis, prescription of steroids, and use of conventional glucose-based PD solution and icodextrin PD solution (Baxter), were obtained from the medical records. A diagnosis of EPS in this study was made based on the clinical features of gastrointestinal obstruction due to bowel obstruction and features of encapsulation with peritoneal fibrosis, according to the definition of the International Society for Peritoneal Dialysis guidelines/recommendations^27^.

Obtained tissues were fixed overnight in 10% buffered formalin and then routinely processed for light microscopy. For assessment of vasculopathy, the lower luminal diameter to vessel diameter (L/V) ratio was determined as described previously^18,25^. Immunostaining for CD31 (JC/70A; Dako, Glostrup, Denmark) and CD68 (PGM 1; Dako) was conducted as described previously^19,26^. Expression of HABP was examined using the same procedures described above for rodent tissues.

### Statistical analyses

Statistical analyses were conducted using SPSS version 25.0 software (IBM, Armonk, NY). Continuous variables were expressed as mean ± standard deviation or median with interquartile range, as appropriate, and categorical variables were expressed as number (proportion). To examine differences between groups, the Student’s *t*-test or Mann–Whitney *U* test was conducted for comparisons of normally distributed or non-parametric data, respectively. Fisher’s exact test was performed for categorical variables. One-way analysis of variance followed by Tukey’s test or the Kruskal–Wallis multiple comparison test was conducted for evaluations between three or more groups. Significance was defined at the level of *p* < 0.05.

## Results

### Effects of hyaluronidase on peritoneal membrane function

We first investigated the effect of degradation of hyaluronan on peritoneal vascular endothelial cells using hyaluronidase or deactivated hyaluronidase (Fig. 1A). Serum hyaluronan levels were significantly increased and hyaluronan expression decreased in the hyaluronidase-treated group but not in the control or deactivated hyaluronidase–treated groups (Fig. 1B–D). These findings indicate that hyaluronidase effectively degraded hyaluronan on the peritoneal vessels. Hyaluronan expression on mesothelial cells was detected and found to be preserved after hyaluronidase treatment (Supplementary Fig. S1). Protein and albumin leakage into the dialysate was significantly increased in the hyaluronidase group compared with the control and deactivated hyaluronidase–treated groups. The D/P β2MG (molecular weight, MW 11,000) ratio was increased in the hyaluronidase-treated group; however, the D/P urea (MW 60) ratio, D2/D0 glucose (MW 180) ratio, and net ultrafiltration (data not shown) were not changed (Fig. 1B). Pathologically, vascular endothelial cells assessed by LEL staining and expression of heparan sulfate, a core protein of the glycocalyx, in CD31-positive blood vessels were not altered by hyaluronidase treatment (Fig. 1C and D, Supplementary Fig. S2). In addition, expression of VE-cadherin, a major protein component of the adherens junction, on CD31-stained sections was also unchanged (Supplementary Fig. S3).

In order to confirm the peritoneal permeability of albumin, we administered EB, a dye that has a high affinity for albumin. Significant EB leakage into the dialysate was observed (Fig. 2), suggesting that albumin leakage was greater following treatment with hyaluronidase. These findings indicate that degradation of hyaluronan of the glycocalyx induced leakage of macromolecules such as proteins, including albumin and β2MG, into the dialysate but not the leakage of small molecules such as urea or glucose.

### Monitoring of hyaluronan expression and peritoneal membrane transport after intraperitoneal administration of hyaluronidase

After loss of peritoneal hyaluronan due to hyaluronidase treatment, changes in peritoneal permeability and hyaluronan expression on the peritoneum were examined (Fig. 3A). Protein leakage into the dialysate decreased and returned to the control levels by 48 h. Increased peritoneal membrane transport of β2MG at 2 h was also gradually decreased and returned to the control level by 48 h. Peritoneal solute transport parameters, including D/P urea and D2/D0 glucose ratios, were unchanged (Fig. 3B). Peritoneal hyaluronan expression assessed by HABP staining also recovered to control levels by 48 h (Fig. 3C and D). LEL expression was not different at 24 and 48 h. Serial changes of hyaluronan expression and peritoneal membrane transport after combing with Fig. 1 (2 h after hyaluronidase treatment) are shown in Supplementary Fig. S4.

### Expression of hyaluronan on human peritoneal membrane

We further investigated the expression of hyaluronan using human peritoneal membrane biopsy samples. First, we compared hyaluronan expression on the peritoneal membrane after long-term treatment (> 4 years) with conventional solutions or low-GDP, pH-neutral solutions. Previously, we reported that endothelial injuries, so called vasculopathies, are pronounced in patients treated with conventional solutions^18,19,26^. We expected that loss of hyaluronan would be enhanced after long-term PD treatment; therefore, peritoneal biopsy samples collected after long-term treatment were evaluated (Conventional-solution group: n = 24; pH-Neutral group: n = 22; Control group: n = 5). Patient characteristics are shown in Table 1. There were no differences in age, sex, primary kidney diseases, PD duration, peritonitis rates, D/P Cr, or use of icodextrin solutions between the groups. Low occurrence rates of peritonitis enabled assessment of the changes in hyaluronan expression due to treatment with the various dialysates. Expression of hyaluronan assessed based on HABP positivity was decreased in the conventional solution group and associated with lower L/V ratio. There was a good correlation between L/V ratio and HABP positivity (*r*^*2*^ = 0.524, *p* < 0.0001) (Fig. 4B and C). There were no significant differences in the number of CD31-positive vessels or CD68-positive macrophages between the groups (Fig. 4C and D).

**Table 1:** Peritoneal membrane biopsy samples from patients treated with conventional solutions and low-GDP, pH-neutral solutions to evaluate HABP expression.

	Neutral solution	Conventional solution	*p* value	
	(n = 22)	(n = 24)		
*Clinical factor*				
Age (y)	61.2 ± 13.1	60.5 ± 8.90	0.846	†
Male, n (%)	17 (77.3)	12 (50.0)	0.056	*
*Primary kidney disease*				
Chronic glomerulonephritis, n (%)	12 (54.5)	10 (41.7)	0.242	*
Diabetes nephropathy, n (%)	4 (18.2)	3 (12.5)		
PD duration (months)	81 (66.0–106.0)	91 (67.0–116.5)	0.367	‡
Peritonitis rate (patient-1 × year-1)	0.0 (0.00–0.30)	0.1 (0.00–0.19)	0.619	‡
D/P Cr	0.62 ± 0.14	0.70 ± 0.15	0.061	†
Use of icodextrin, n (%)	16 (76.2)	11 (45.8)	0.038	*
*Reason for withdrawal from PD*				
Dialysis failure/UF failure, n (%)	7 (31.8)	13 (54.2)	0.152	*
Prevention of EPS, n (%)	11 (50.0)	8 (33.3)		

Neutral solution: PD patients treated with low-GDP, pH-neutral solution for more than 4 years. Conventional solution: PD patients treated with conventional solution.

Data are presented as number (percentage) or mean ± SD. *SD* standard deviation.

*n (%), Fisher's exact test, †mean ± SD, Student *t*-test, ‡median (IQR), Mann–Whitney *U* test.

Next, we examined the expression of hyaluronan in EPS cases (Table 2). We previously reported that the pathology of EPS differs between patients treated with conventional versus pH-neutral solutions and that vasculopathy assessed based on the L/V ratio is more severe in patients treated with conventional solutions versus pH-neutral solutions. In addition, the difference in L/V ratio was more pronounced in cases of EPS recurrence. In contrast, in the pH-neutral solution group, peritonitis-induced adhesions appeared to be an important factor^26^. In the present study, there were no differences between groups in terms of age and primary kidney diseases. As in previous reports, in the pH-neutral solution group, the duration of PD treatment was shorter, and 89% of cases had a peritonitis episode within a few months before the occurrence of EPS. Expression of hyaluronan was lower in the EPS-conventional solution group and associated with lower L/V ratio. Importantly, expression of hyaluronan declined even though the L/V ratio was relatively high (Fig. 5B and C), suggesting that a decrease in hyaluronan expression may play an important role in the development of EPS in patients treated with conventional solutions. HS expression tended to be lower in the EPS-conventional solution group than in the EPS-neutral group (Fig. 5B and C). The extent of HS loss was lower than the extent of hyaluronan loss (Supplementary Fig. S5). The number of CD31-positive vessels was higher in EPS patients treated with neutral solutions than in those treated with conventional solutions (Fig. 5C and D).

**Table 2:** Peritoneal membrane samples taken from EPS patients at the time of enterolysis surgery.

	EPS-Neutral	EPS-Conventional	p-value	
	(n = 9)	(n = 61)		
*Clinical factors*				
Age (y)	63 (57.0–68.0)	56 (49.0–62.0)	0.124	‡
*Primary kidney disease*				
Chronic glomerulonephritis, n (%)	7 (77.8)	56 (91.8)	0.338	*
Diabetes nephropathy, n (%)	2 (22.2)	3 (4.9)		
PD duration (months)	63 (29.8–83.0)	136 (108.0–171.9)	< 0.001	‡
Peritonitis within a few months, n (%)	8 (88.9)	15 (24.6)	< 0.001	*

EPS-Neutral: EPS cases after use of low-GDP, pH-neutral solution.

EPS-Conventional: EPS cases after use of conventional solution.

Data are presented as number (percentage) or mean ± SD. *SD* standard deviation.

*n (%), Fisher's exact test, †mean ± SD, Student *t*-test, ‡median (IQR), Mann–Whitney *U* test.

We also examined the expression of hyaluronan in patients with acute peritonitis. There were no differences in hyaluronan expression in the peritoneum in patients treated with pH-neutral solutions, with or without peritonitis (Supplementary Fig. S6). In the CG model, a non-infectious peritonitis–induced fibrosis model^28^, expression of hyaluronan and heparan sulfate was also preserved in CD31-positive blood vessels of the peritoneum (Supplementary Fig. S7), as observed in human peritonitis.

## Discussion

The glycocalyx plays a crucial role in vascular pathology. Damage to the glycocalyx is related to a variety of conditions, such as sepsis^29–31^, renal failure^10,32^, and diabetes^13,17,31,33,34^. Digestion of hyaluronan on glomerular endothelial cells by administration of hyaluronidase was recently shown to induce proteinuria ex vivo, which can exclude the influence of circulating mediators, and this has also been shown in vivo^15,16^. In vascular endothelial growth factor (VEGF) signaling inhibition–related glomerular diseases, such as thrombotic microangiopathy associated with anti-VEGF therapy or pre-eclampsia^35,36^, glomerular hyaluronan is diminished in association with proteinuria^15,37,38^. Loss of hyaluronan in the glycocalyx also plays a role in the development of diabetic nephropathy^17,33^. These findings indicate that degradation of hyaluronan in the glomerulus leads to proteinuria and structural damage^15–17^. These phenomena led us to hypothesize that the loss of hyaluronan induces leakage of large molecules and protein into the dialysate, leading to a number of related complications in PD patients.

**Figure 6:**
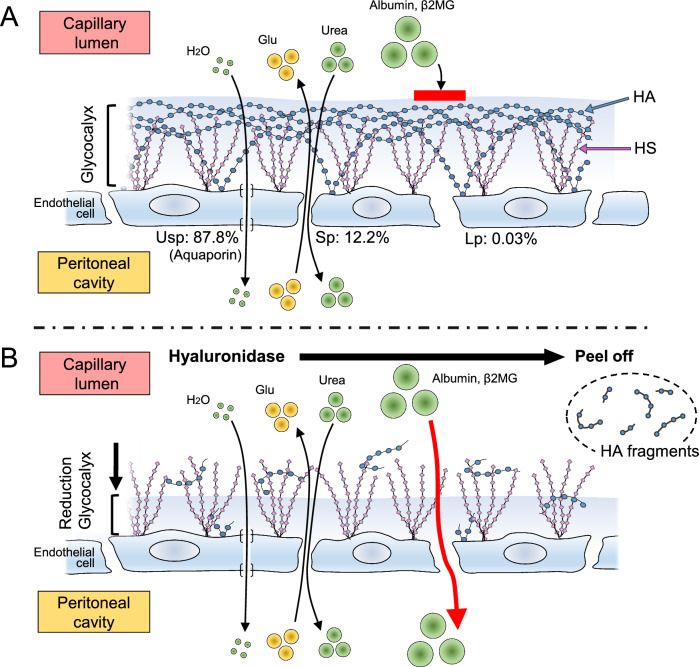
Schematic diagram of the role of hyaluronan in peritoneal transport. (**A**): The surface of glomerular endothelial cells of peritoneal vessels is covered with the glycocalyx, a bioactive gel-like layer composed of heparan sulfate (HS), hyaluronan (HA), and associated proteins. Water and small solutes, such as glucose, can pass between endothelial cells through ultra-small pores or small pores, respectively. The ratios of the pore areas of ultra-small (Usp), small (Sp), and large pores (Lp) are reportedly 87.8%, 12.2%, and 0.03%, respectively (43). (**B**): HA is degraded into fragments by hyaluronidase treatment. After HA digestion, large molecules, such as albumin and β2MG, can leak into the dialysate through large pores. Glu: glucose; β2MG: beta-2 microglobulin.

Only a few reports have been published to date concerning the expression of glycocalyx in the peritoneal membrane in PD^39^. To the best of our knowledge, there are no reports describing the roles and expression of hyaluronan in the endothelial glycocalyx on the peritoneal membrane. The present study showed that hyaluronan in the glycocalyx on peritoneal vascular endothelial cells plays a crucial role in preventing leakage of protein into the dialysate (Figs. 1, 2, 3). Previously, it was difficult to identify the loss of hyaluronan and glycocalyx, which was judged by measuring thickness using sophisticated techniques^12,40–42^. The present study successfully demonstrated the presence of hyaluronan in the vascular endothelial surface layer using biotin-labeled HABP on paraffin-embedded sections. The present study also demonstrated that peritoneal permeability for large molecules, such as proteins, albumin, and β2MG, increases after degradation of hyaluronan but improves after recovery of hyaluronan (Figs. 1 and 3). Our data showed that hyaluronan itself plays an important role in preventing the leakage of macromolecules, including albumin and β2MG (Fig. 6). These phenomena are similar to proteinuria induced by degradation of hyaluronan in glomerular endothelial cell injury.

It is widely known that there are three sizes of pores between peritoneal vascular endothelial cells. Ultra-small pores (2.5 Å in diameter) are formed by aquaporin-1, which is involved in water passage. Small pores (diameter 45 Å) and large pores (diameter 250 Å) are involved in the passage of small solutes or large molecules^43^ (Fig. 6). The number of large pores is very low, and the ratios of the pore areas of ultra-small, small, and large pores are reportedly 87.8%, 12.2%, and 0.03%, respectively^43^. One function of peritoneal glycocalyx is thought to be regulation of the transport of macromolecules across the endothelial layer rather than small solutes^44^. Our data indicate that hyaluronan regulates macromolecule transport across endothelial cells, as shown in Fig. 6. The glycocalyx does not appear to be a barrier to small molecules.

Previous research in PD primarily focused on mesothelial cell–derived hyaluronan. Hyaluronan is known to function as a lubricant, a component of the mesothelial glycocalyx, which protects the mesothelium from abrasion and adhesion^45,46^. Intraperitoneal administration of high-molecular-weight hyaluronan or PD solution containing hyaluronic acid has been attempted, but the results have been controversial, and there was no interpretation or discussion in reports of those studies regarding the role of hyaluronan in the glycocalyx on vascular endothelial cells^47,48^.

In this study, we measured the expression of hyaluronan in human peritoneal biopsy samples to investigate the possible role of hyaluronan in disease conditions involving protein leakage into PD fluid (Figs. 4 and 5). Low-GDP, pH-neutral PD solutions reportedly ameliorate structural and functional injuries to a greater degree than conventional solutions^18,19,49^. We previously reported that severe vasculopathy involving damage to vascular endothelial cells can lead to leakage of plasma and fibrin, resulting in intestinal adhesions^18,26^. These effects are more pronounced with the use of conventional solutions, resulting in a high incidence of EPS^18,26^. We also recently reported that peritoneal vascular endothelial HS assessed using three antibodies that identify different domains of HS and fucose-containing sugar chains is better preserved during exposure to low-GDP, pH-neutral solutions than conventional solutions^39^. The present human data indicate that loss of hyaluronan is more often observed in patients receiving conventional solutions. Our data support the hypothesis that degradation of the glycocalyx, which is more pronounced with conventional solutions, results in greater protein leakage, leading to the development of EPS (Supplementary Fig. S8).

In contrast, in peritonitis, hyaluronan is preserved in the vessels in human and animal CG models of peritonitis-related fibrosis^28^ (Supplementary Figs. S4 and S5). A large amount of protein can leak into the dialysate during peritonitis. The altered permeability is reportedly due to endotoxin and complement activation, prostaglandins, IL-6, TNFα, and nitric oxide^50^. Changes in hyaluronan were not observed, suggesting that chemical mediators are the main cause of protein loss in peritonitis.

The present study has some limitations. The number of patient samples involving EPS-neutral solutions was small, because the occurrence rate of EPS is very low in treatment with pH-neutral solutions in Japan^51^. Previous studies have suggested that peritoneal membrane damage decreases after changing to low-GDP, pH-neutral solutions^18,19,49^. It was difficult to investigate the exact mechanism of hyaluronan reduction in endothelial cells of the EPS conventional group due to the limited size of human peritoneal tissue samples. All patients in this study were Japanese, so it may be difficult to generalize these data to other populations.

In summary, our data suggest that hyaluronan, a component of the glycocalyx, plays an important role in peritoneal membrane transport of macromolecules. Loss of hyaluronan in the glycocalyx may induce leakage of proteins and macromolecules, ultimately leading to the development of EPS (Supplementary Fig. S6).

### Supplementary Information

Supplementary Information.

## Data Availability

The data may be shared upon reasonable request to the corresponding author, if the request is accepted by the ethics committees.

## Abbreviations

PD: Peritoneal dialysis
EPS: Encapsulating peritoneal sclerosis
GDP: Glucose degradation product
PET: Peritoneal equilibration test
PDF: Peritoneal dialysis fluid
β2MG: β2 Microglobulin
D/P β2MG: Dialysate-to-plasma β2 microglobulin ratio
D/P creatinine: Dialysate-to-plasma creatinine ratio
D/P urea: Dialysate-to-plasma urea ratio
D2/D0 glucose: Ratio of dialysate glucose at 2 h of dwell time to dialysis glucose at baseline
EB: Evans Blue
HS: Heparan sulfate
L/V ratio: Luminal diameter to vessel diameter
VEGF: Vascular endothelial growth factor
CG: Chlorhexidine gluconate
IL: Interleukin
TNFα: Tumor necrosis factor–alpha
