# Supplementary material for: Role of endothelial hyaluronan in peritoneal membrane transport and disease conditions during peritoneal dialysis
Source: Sci Rep. 2024 Mar 28;14:7412. doi: 10.1038/s41598-024-58148-x (PMC10978880; doi:10.1038/s41598-024-58148-x)
Supplement: Supplementary file 1 — Supplementary Information. [file 41598_2024_58148_MOESM1_ESM.pdf]

Role of endothelial hyaluronan in peritoneal membrane transport and disease conditions during peritoneal dialysis

<u>Supplementary materials</u>

Keisuke Kamiya, Naoyuki Hatayama, Mitsuhiro Tawada, Akimasa Asai, Mai Yamauchi, Hiroshi Kinashi, Shunnosuke Kunoki, Makoto Yamaguchi, Masashi Mizuno, Yasuhiro Suzuki, Masataka Banshodani, Takuji Ishimoto, Munekazu Naito, Hideki Kawanishi, Yasuhiko Ito

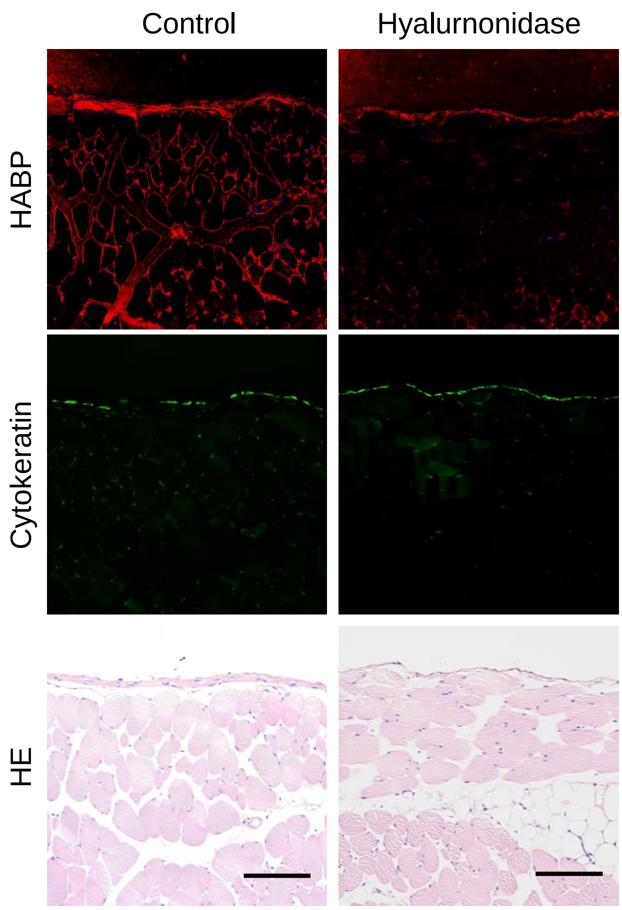

Supplementary Figure S1. Expression of hyaluronan and cytokeratin staining on the serial sections.

Hyaluronan expression was detected on mesothelial cells and found to be preserved after hyaluronidase treatment. Scale bars = 50 μm

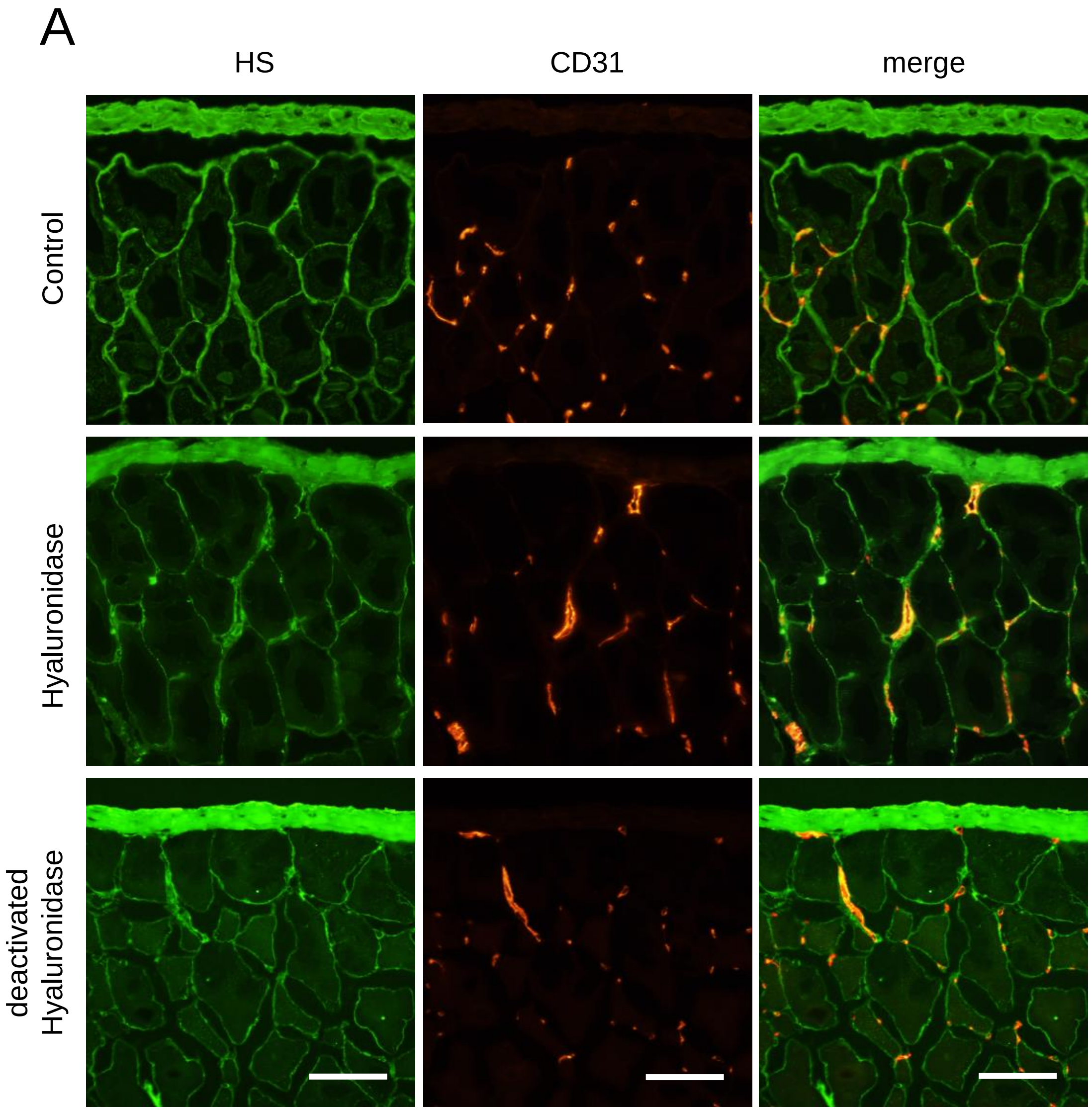

Supplementary Figure S2A. Double staining for Heparan Sulfate (HS) and CD31. Double-staining for HS, core protein of the endothelial glycocalyx, and CD31 on mouse frozen tissues was conducted to assess changes of HS expression in the vessels after hyaluronidase treatment.
Scale bars = 50 μm

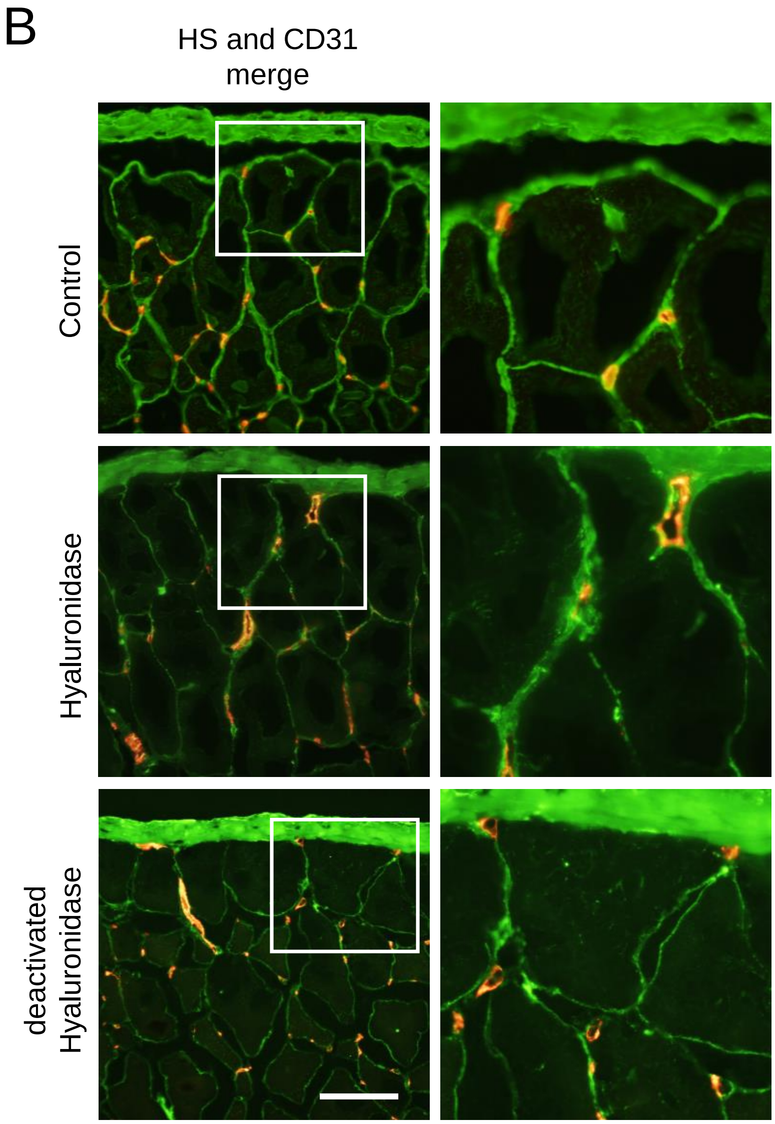

Supplementary Figure S2B. Double staining for Heparan Sulfate (HS) and CD31. HS positivity (HS-positive vessels / CD31-positive vessels) were assessed on the same section. Expression of HS in the CD31 positive vessels did not change after hyaluronidase or deactivated hyaluronidase treatment. The results are shown in **Fig. 1D**.
Scale bars = 50 μm

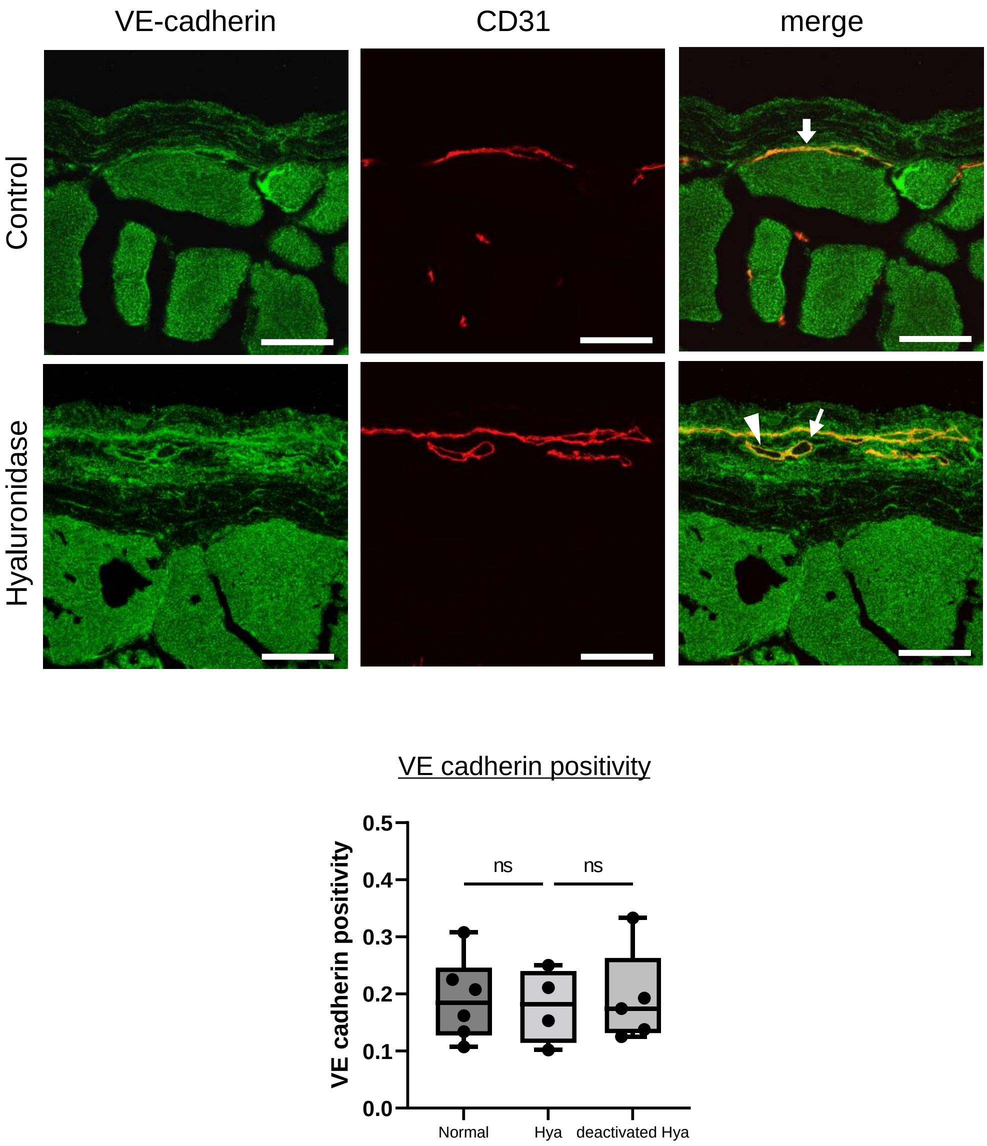

Supplementary Figure S3. Double staining for VE-cadherin and CD31. VE-cadherin is a major protein component of adherens junctions of the vessels. VE cadherin-positivity (VE cadherin-positive vessels / CD31-positive vessels) was assessed on the same sections. VE cadherin-positivity was not changed between the groups. Arrows and arrowhead indicate the double positive vessels. Scale bars = 50 µm

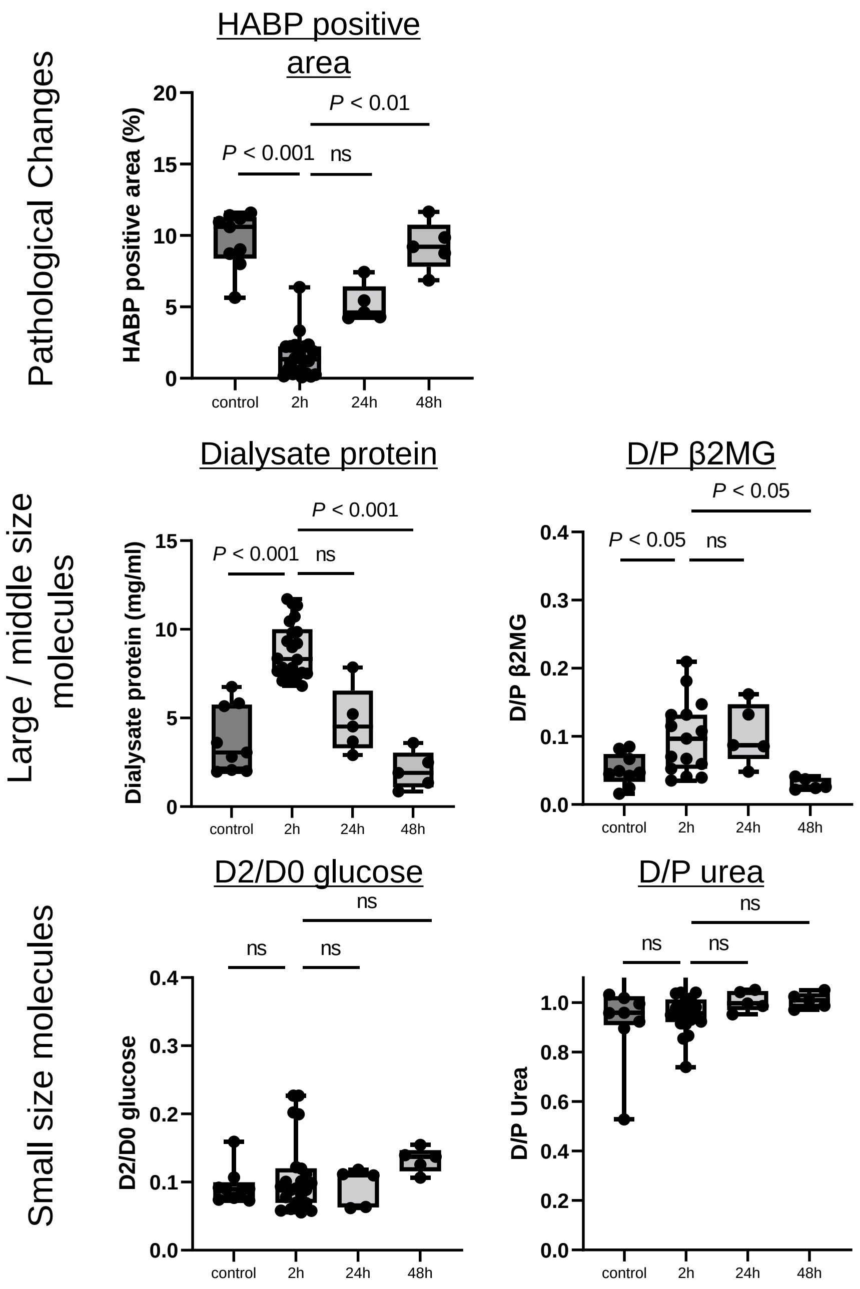

Supplementary Figure S4. Serial changes of hyaluronan expression and peritoneal membrane transport after intraperitoneal administration of hyaluronidase

Serial changes of hyaluronan expression and peritoneal membrane transport after combing with **Fig. 1B** and **D** are shown. Expression of hyaluronan as assessed by HABP stain recovered at 48 h after digestion with hyaluronidase. Increased leakage of protein and β2MG into the dialysate also returned to the control levels at 48 h. Small solute transport was unchanged during the experiments.

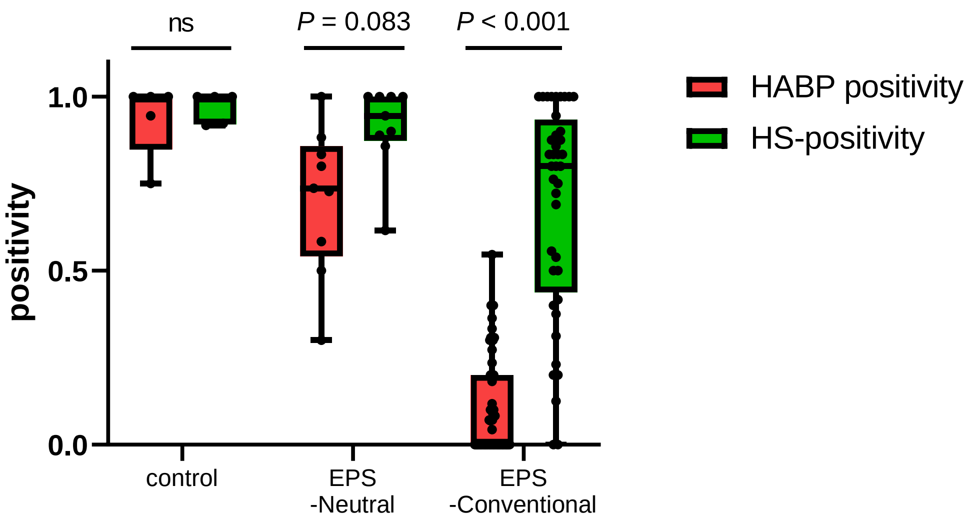

Supplementary Figure S5. Comparison of hyaluronan and HS expression in human EPS peritoneal membrane

The extent of HS loss was lower than the extent of hyaluronan loss.

A

	Non-peritonitis-Neutral (n=41)	Peritonitis-Neutral (n=7)	*P*-value
Clinical factors			
Age (y)	64 (54.0 - 70.0)	56 (45.0 - 62.0)	0.183 ‡
Male, n (%)	33 (80.5)	6 (85.7)	0.608 *
Primary kidney disease			
Chronic glomerulonephritis, n (%)	10 (24.4)	3 (42.9)	0.416 *
Diabetes nephropathy, n (%)	18 (43.9)	3 (42.9)	
PD duration (month)	24 (15.0 - 48.0)	21 (10.0 - 26.5)	0.164 ‡
Use of icodextrin, n (%)	33 (80.5)	3 (42.9)	0.055 *

B

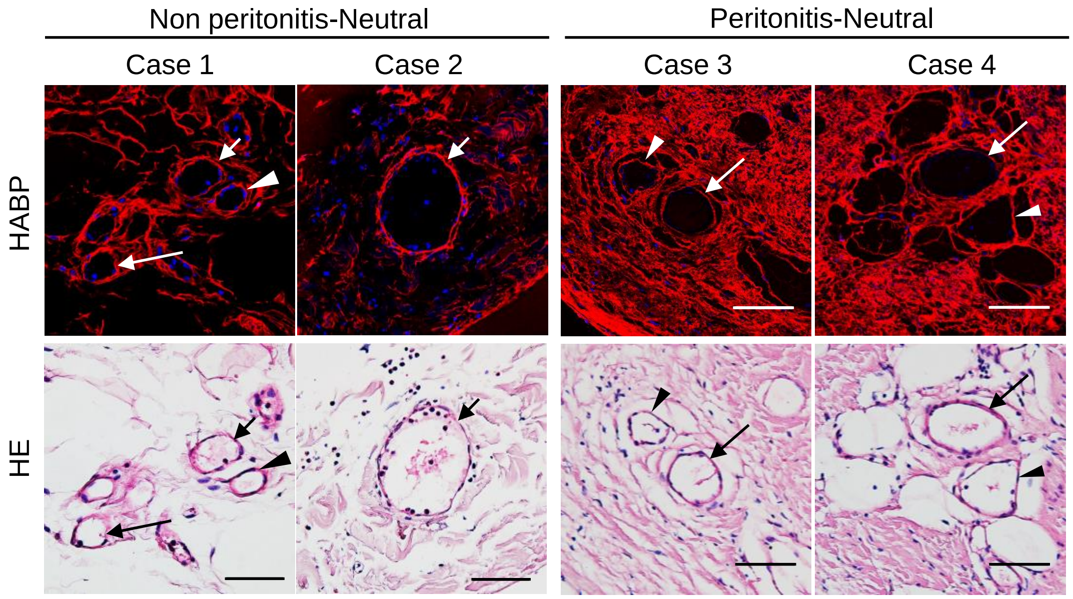

C

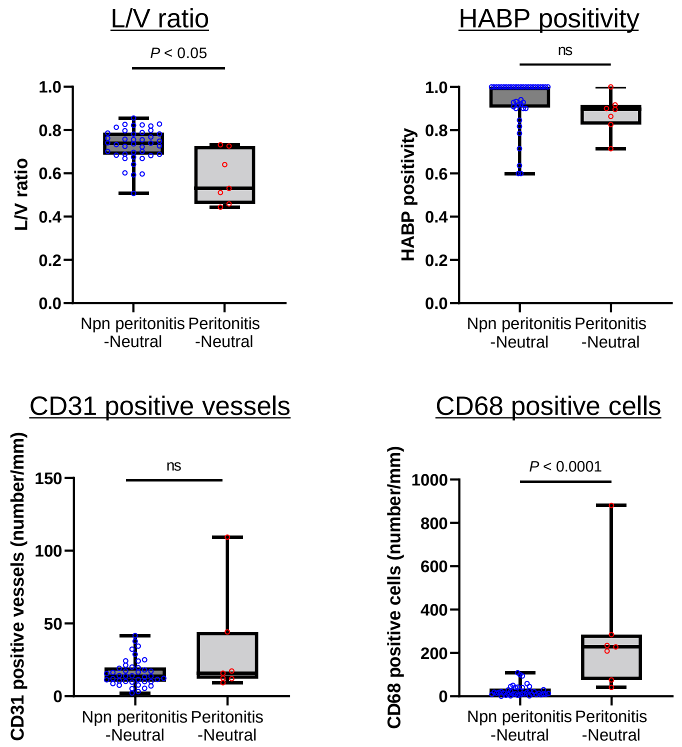

Supplementary Figure 6. Expression of hyaluronan in human PD-related peritonitis

Supplementary Figure S6. Expression of hyaluronan in human PD-related Peritonitis

Changes of expression of hyaluronan in peritonitis were assessed using the samples of the patients who were treated with low GDP, pH neutral solutions. There were no differences in hyaluronan expression assessed by HABP stain between peritonitis (Peritonitis-Neutral group) and non-peritonitis conditions (Non-peritonitis-Neutral group). As expected, the number of CD 68 positive cells were higher in Peritonitis-Neutral group than that in Non-peritonitis-Neutral group. Representative 2 cases are shown. Arrows and arrowheads indicate the same vessels of each case.
*n (%), Fisher's exact test, †mean±SD, Student t-test, ‡median (IQR), Mann-Whitney's U-test
L/V ratio: luminal diameter to vessel diameter; HABP: hyaluronan-binding protein; n.s.: not significant. Scale bars = 100 μm

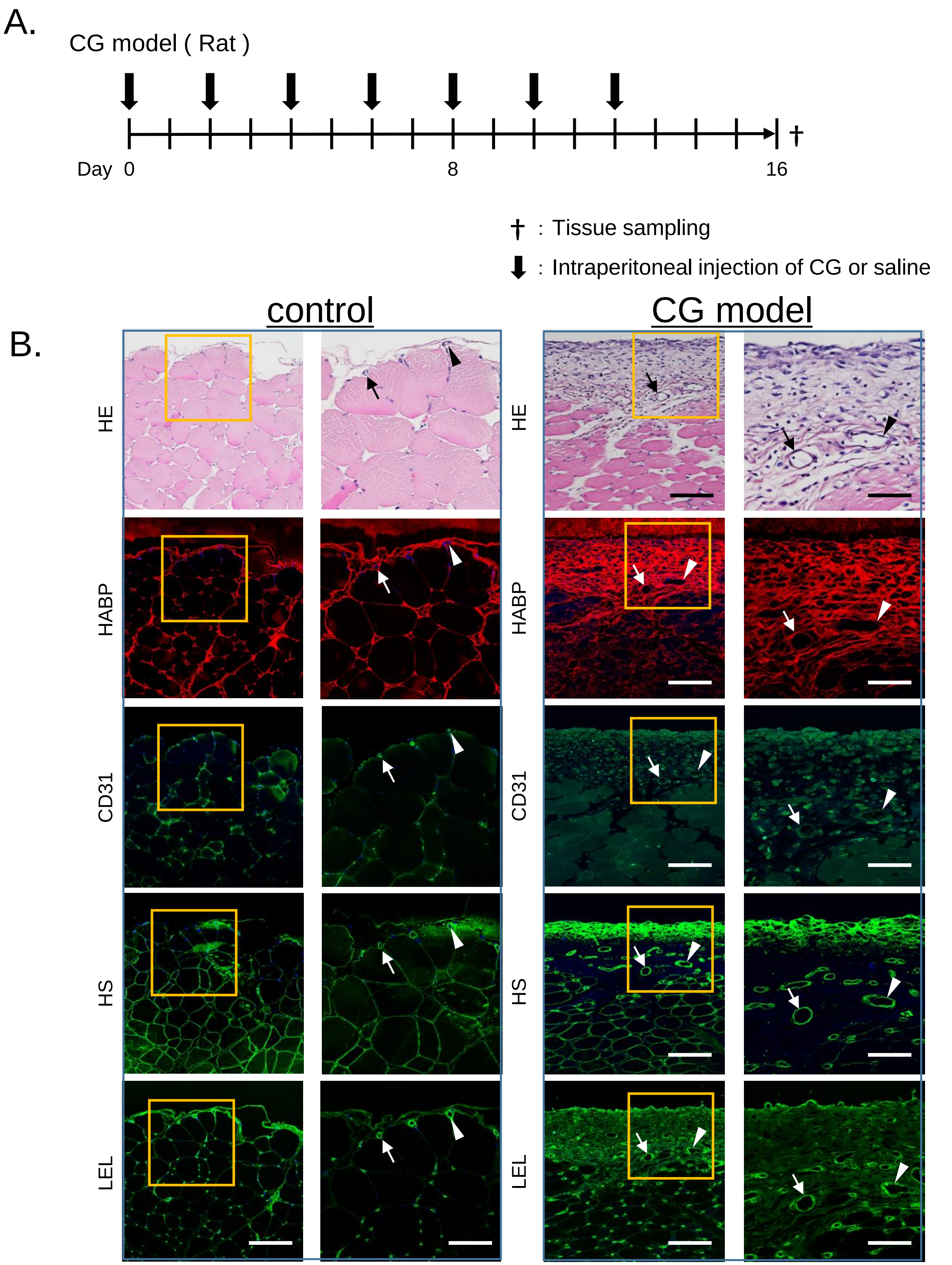

Supplementary Figure S7-A, B. Expression of hyaluronan in the chlorhexidine-induced peritoneal injury model

C

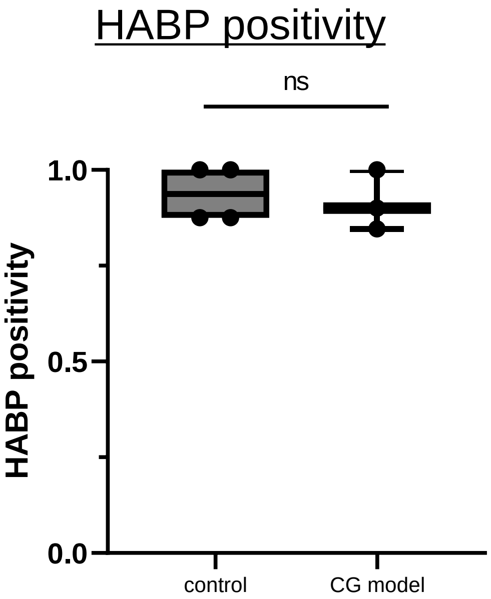

Supplementary Figure S7. Expression of hyaluronan in the chlorhexidine-induced peritoneal injury model

A. To investigate the expression of peritoneal vascular endothelial hyaluronan in chlorhexidine gluconate (CG)-induced model, a peritonitis induced fibrosis model (28), 8-week-old male Sprague-Dawley rats received intraperitoneal injection of CG (Wako Pure Chemicals) (3 ml/200 g body weight) dissolved in saline with 10% ethanol (Wako Pure Chemicals) or every other day until day 12 (n= 4). Rats were euthanized and parietal peritoneal samples were taken on day 14, and subsequently pathological analyses were conducted. Rats that had received saline with 10% ethanol were used as controls (n= 4) (28).
B, C. Analyses on the serial sections indicate the hyaluronate expression assessed by HABP staining and HS were preserved in the vessels of CG model. CD31 and LEL by arrow and arrow head indicate the same vascular endothelial cells. The picture of right column shows a large magnification of square in each left picture. There was no difference in HABP positivity between the groups.
Arrow and arrow head indicate the same vessels.
Scale bars = 100 (left column) and 50 (right column) µm.

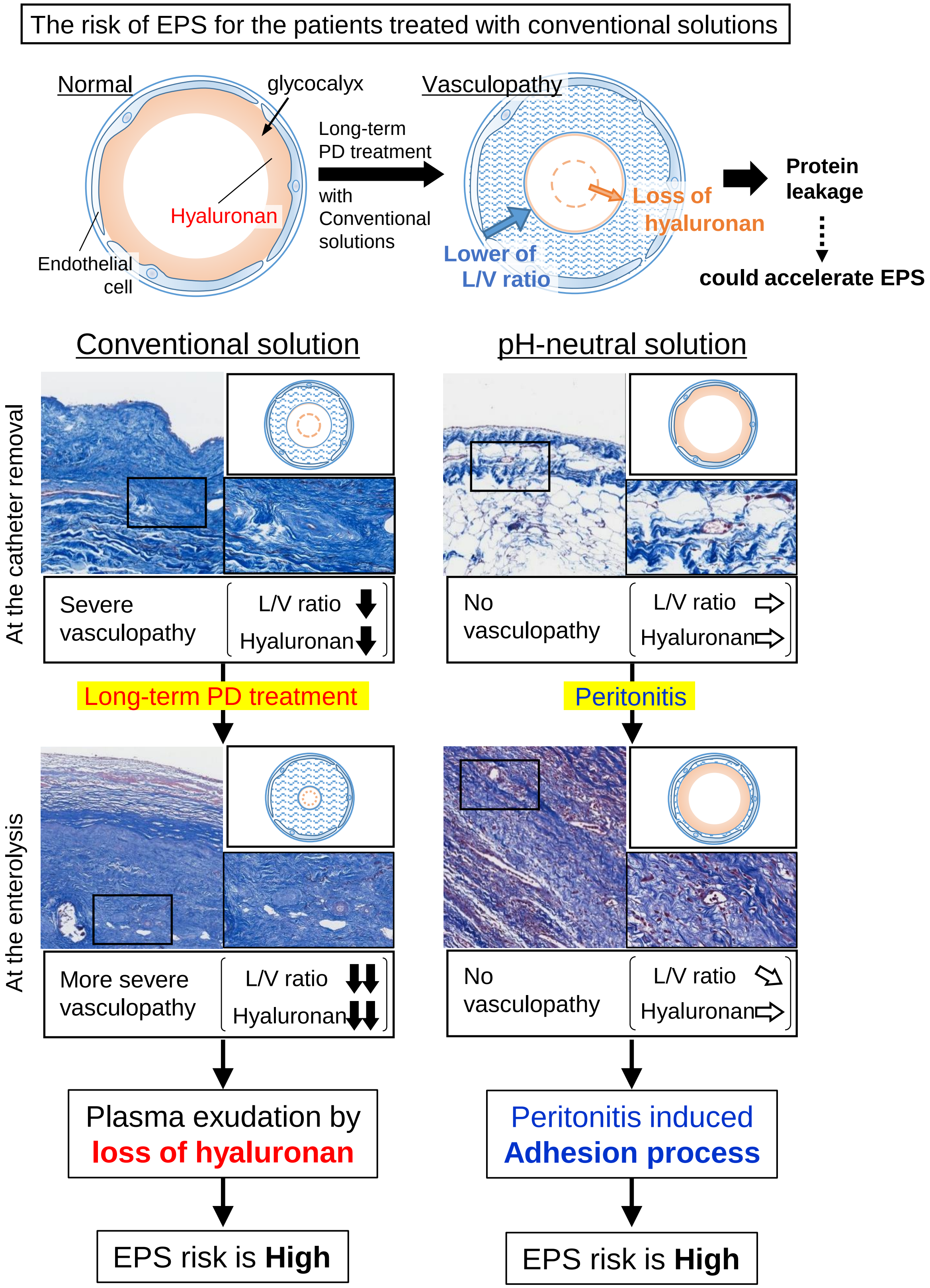

Supplementary Figure 8. A hypothesis of the roles of hyaluronan in the development of EPS.

Vascular endothelial cells are covered with glycocalyx comprising heparan sulfate, hyaluronan and associated proteins. This study indicates that hyaluronan in the endothelial glycocalyx is involved in protecting against leakage of macromolecules including protein (**Figs. 1-3**) as reported in the glomerulus (refs. 15-17).

Vasculopathy reportedly progress significantly in patients treated with conventional solution when compared with patients treated with pH-neutral low-GDP solution (refs 19, 49), and severe vasculopathy could be a predictor for the development of EPS (ref. 18). In the EPS cases, vasculopathy assessed by L/V ratio was more severe in the conventional solution group than in the pH-neutral solution group (**Fig. 5**) (ref. 26). In addition, L/V ratio was more pronounced in the recurrence cases of EPS (ref. 26). In contrast in the pH-neutral solution group, peritonitis induced adhesion seemed to be an important factor (ref. 26), and hyaluronan was preserved (**Fig. 5**). We observed a good correlation between L/V ratio and HABP positivity (**Figs. 4B and C**). Loss of hyaluronan is apparent in the conventional solutions groups (**Figs. 4C and 5C**), even though the L/V ratio was relatively preserved **(Fig. 5C**). Taken together, we propose a hypothesis that degradation of hyaluronan which is more pronounced with conventional solutions can enhance the protein leakage leading to development of EPS.

Supplementary Figure S8. A hypothesis of the roles of hyaluronan in the development of EPS.

Antibody	Company
Human Hyaluronan Binding Protein, Biotin	Hokudo, Sapporo, Japan
Mouse anti-Human heparan sulfate (10E4 epitope) antibody	United States Biological, Salem, MA
FITC-labeled goat anti-mouse IgM	Southern Biotech, Birmingham, AL
Alexa 555 labeled goat anti-rat IgG	Thermo Fisher Scientific, Waltham, MA
Alexa 555 conjugate streptavidin	Thermo Fisher Scientific, Waltham, MA
Alexa 488 labeled goat anti-rabbit IgG	Thermo Fisher Scientific, Waltham, MA
diamidino-2-phenylindole (DAPI)	Dojindo, Kumamoto, Japan
Rabbit anti-VE Cadherin antibody	Bioss Antibodies, Woburn, MA
Goat Anti-Rabbit IgG (H+L)	Southern Biotech, Birmingham, AL
Rat anti-mouse CD31 antibody	Merck, Darmstadt, Germany
Rabbit anti-rat CD31 antibody	Proteintech, Rosemont, USA
Anti-CD31 antibody (JC/70A)	Dako, Glostrup, Denmark
Anti-CD68 antibody (PGM1)	Dako, Glostrup, Denmark
Anti-pan Cytokeratin, FITC antibody	Sigma-Aldrich, St.Louis, MO

Lectin	Company
Tomato fluorescein lycopersicon esculentum lectin	Vector Laboratories, Burlingame, USA

ELISA kit	Company
BCA Protein Assay Kit	Thermo Fisher Scientific, Waltham, MA
Creatinine ELISA assay kit	FUJIFILM Wako Pure Chemical, Osaka, Japan
Hyaluronan ELISA assay kit	PG Research, Tokyo, Japan
LBIS Mouse Urinary Albumin Assay Kit (S-type)	FUJIFILM Wako Shibayagi, Gunma, Japan
Mouse Beta-2-Microglobulin ELISA Kit	Abcam, Cambrudge, UK

Supplementary Table 1. List of antibodies and ELISA kits used
